# A Review of Colorectal Cancer Detection Modalities, Stool DNA, and Fecal Immunochemistry Testing in Adults Over the Age of 50

**DOI:** 10.7759/cureus.931

**Published:** 2016-12-16

**Authors:** Tyler Janz, Karen Lu, Michael R Povlow, Brittany Urso

**Affiliations:** 1 Medical Student, University of Central Florida College of Medicine

**Keywords:** colorectal cancer, crc, stool dna testing, multitarget dna testing, sdna, fecal immunochemisty testing, fit, colonoscopy, colorectal screening

## Abstract

Colorectal cancer (CRC) is the third leading cause of cancer death in the United States. Recently, more focus has been placed on developing effective screening tools to detect the presence of both precancerous and cancerous lesions present in the colon and rectum. Colonoscopy has been well established as the gold standard of the colon and rectal cancer screening. However, not all patients are willing to undergo a colonoscopy due to the procedure’s invasive nature. Non-invasive screening methods have been developed to appeal to patients who refuse colonoscopy. Fecal occult blood tests have long been used by physicians, in addition to colonoscopy, in an effort to screen for CRC. New screening methods, such as fecal immunochemical test (FIT) and stool DNA (sDNA) testing, have been developed as a more sensitive screening measure to attempt to accurately screen patients who have precancerous or cancerous colorectal lesions. This article compares CRC screening techniques through literature review in order to determine which tests offer the most sensitive detection of CRC and precancerous lesions in average-risk adults over the age of 50 years old. Through this review, it can be seen that sDNA is more sensitive than FIT in detecting all stages of CRC, as well as precancerous lesions.

## Introduction and background

Colorectal cancer (CRC) contributes to 8% of all new cancer cases in the United States annually. There have been 134,490 estimated new CRC cases and 49,190 estimated deaths from CRC in 2016. People aged 65 - 74 years old are most often diagnosed with CRC, with the median age of diagnosis of 68 years old [1]. Currently, colon and rectal cancers are the third most common cause of new cancer in the United States (US) as well as the third deadliest type of cancer [2]. Therefore, it is critical for physicians and the general population to understand the pathogenesis and screening methods for the detection of colon cancer.

The US Preventive Services Task Force (USPSTF) recommends all adults between the ages of 50 - 75 years old undergo screening for CRC. In June 2016, the USPSTF updated these screening guidelines to include fecal immunochemical test (FIT), stool DNA test (sDNA), and flexible sigmoidoscopy with FIT, in addition to the previously recommended guaiac-based fecal occult blood test (gFOBT), colonoscopy, CT colonography, and flexible sigmoidoscopy [3].

Detecting CRC begins before cancer has developed. Knowledge of the pathogenesis of colon cancer is critical to understanding the different types of screening methods. The main pathogenesis for the development of colon cancer relies upon a stepwise progression in the acquisition of several chromosome mutations. Chromosomal instability in the adenomatous polyposis coli (APC) gene, the KRAS oncogene, and the p53 tumor suppressor genes all play an important role in the development of colon cancer [4]. APC protein is often the first mutation to develop. Mutation or loss of APC causes, more commonly, inherited forms of CRC. However, dual deletions or mutations can prompt sporadic adenoma development. As the early adenoma continues to grow, it begins to accumulate mutations. The methylation of KRAS is found early on, in approximately 13-95% of CRC patients. As the adenoma continues to accumulate mutations, p53 becomes either mutated or deleted, resulting in loss of cell cycle regulation, apoptosis, and response to DNA damage, allowing for the transformation into cancer. p53 mutations are present in 30-60% of CRC patients [5]. Because the development of CRC requires the accumulation of mutations, most CRCs grow slowly over time. The discovery of different types of mutations contributing to the development of CRC is vital to the understanding of creating effective screening methods for CRC. However, new research has shown the development of CRC from serrated sessile polyps (SSPs), which have been shown to grow more rapidly than the adenoma-carcinoma sequence listed above [6].

Adenomatous polyps and SSPs require increased surveillance due to their malignancy potential. On average, less than 5% of adenomas progress to CRC. Those that do are believed to progress to CRC over a seven to 10 year time period. There are two main types of adenomas, villous and tubular. Villous adenomas have the highest malignant potential, make up 5-15% of adenomas, and are very glandular. Tubular adenomas make up about 80% of adenomas, consist of branching adenomatous tissue, and are less likely to progress to CRC [7]. The malignant potential of SSPs is currently under dispute by pathologists. Some pathologists believe that SSPs have a higher rate of progression to CRC than villous adenomas, whereas some believe they have a lower malignant potential [8]. Additionally, SSPs may develop from hyperplastic polyps, which were previously believed to be non-neoplastic in nature [9]. Currently, SSPs are managed like adenomatous polyps and removal is recommended despite the disputed CRC progression rate.

As colorectal cancers grow, the abrasion of passing stool against the cancer causes the release of cells and blood from the lesion into the stool. The blood released mixes with the stool and releases upon defecation. The detection of blood in stool has been a mainstay in the approach to detecting colonic masses. FIT, gFOBT, and sDNA are screening tests which rely on different techniques for the detection of colorectal cancer (Table 1) [10-11]. FIT detects the presence of microscopic amounts of blood present in the stool during defecation. This method is performed via the utilization of antibodies targeted to detect the presence of globin molecules. The antibodies preferably target lower gastrointestinal bleeds, making it ideal for the detection of CRC [10]. This method is not as effective in the detection of upper gastrointestinal bleeds because the hemoglobin undergoes degradation by digestive enzymes, which make the FIT testing antibodies less likely to detect and bind the hemoglobin [10]. sDNA testing, also known as multitarget stool DNA testing or FIT-DNA, also detects eleven different DNA sequences found commonly in colon polyps and CRC [11]. These tests include quantitative molecular assays for KRAS mutations, NDRG4 and BMP3 methylation, and β-actin [12]. Current sDNA tests detect the above-mentioned genes and utilize the same technology of FIT in hemoglobin immunoassays.

**Table 1:** Colorectal Cancer Testing Methods Definitions of gFOBT, FIT, and sDNA tests as defined according to basis and method of each test. gFOBT: guaiac-based fecal occult blood test; FIT: fecal immunochemical test; sDNA: stool DNA testing

gFOBT Testing	FIT Testing	sDNA Testing
Detects the presence or absence of heme in stool.	Detects microscopic blood present in stool via antibodies to globin.	Includes the same properties of FIT testing. Also includes 11 different DNA sequences commonly seen in colon polyps/cancers.

Studies have examined each of these screening tools in regard to the detection of colon cancer. However, very few papers have reviewed the sensitivity and specificities of each screening method in the detection of colon cancer. High sensitivity is the most important factor when evaluating a screening method for colon cancer as this will lead to the further evaluation for colon cancer. The goal of the paper is to compare the colorectal cancer detection sensitivity of FIT, sDNA testing, and gFOBT in patients older than 50 years old with average colorectal cancer risk.

## Review

The 2004 study completed by Imperiale, et al.evaluated 4,404 average-risk, asymptomatic individuals over the age of 50 (Table 2) [4]. The study compared sDNA and gFOBT in the screening of CRC. Subjects were asymptomatic individuals at average risk for CRC. Each subject submitted stool samples for three Hemoccult II tests, stool DNA (sDNA) analysis, and then underwent colonoscopy after all the stool samples were collected. sDNA testing in this study detected 29 of 71 high-grade dysplastic adenomas and invasive cancers (95% CI 40.8 (30.2-52.5)) in comparison to the Hemoccult II, which detected only 10 of 71 high-grade dysplastic adenomas or invasive cancers (95% CI 14.1 (7.8-24.6)). Furthermore, sDNA testing in this study detected 16 of 31 invasive cancers (95% CI 51.6 (34.8-68.0)) in comparison to the Hemoccult II, which detected only four of 31 patients who had either high-grade dysplastic adenomas or invasive cancers (95% CI 12.9 (5.1-28.9)). The study concluded that sDNA testing detected a greater proportion of colorectal neoplasia than compared to Hemoccult II [4].

**Table 2:** sDNA and gFOBT Sensitivity in the Detection of Colorectal High-Grade Dysplastic Adenomas and Invasive Cancers Detection rates and sensitivities of sDNA and gFOBT tests of colorectal high-grade dysplastic adenomas and invasive cancers according to a 2004 study completed by Imperiale, et al. [4] based upon 4,404 average-risk, asymptomatic individuals over the age of 50. gFOBT: guaiac-based fecal occult blood test; sDNA: stool DNA testing

	Testing Method: Detection Rate and Sensitivity	
	sDNA	gFOBT (Hemoccult II)
High-grade dysplastic adenomas and invasive cancers (n = 71)	29 (40.8)	10 (14.1)
Invasive cancers (n = 31)	16 (51)	4 (12.9)

Aliquist, et al. further analyzed the accuracy of sDNA in CRC detection (Table 3) [13]. This study was a blinded, multicenter case-control study of 678 patients. Stool samples were taken from patients with CRC with at least one colorectal adenoma > 1 cm. Controls were archival stools without neoplasia on colonoscopy, matched for age and sex. sDNA testing detected KRAS mutation, α-actin, and hemoglobin, using HemoQuant. Patients were divided randomly into two sets, with the training set containing two-thirds of the patients, and the test set containing one-third of the patients. The study showed that, at a modeled specificity cut-off of 90%, sDNA testing detected 89% of CRC, 62% of adenomas > 1 cm, and 56% of adenomas ≥ 1 cm among the training set. Among the test set, at a modeled specificity cut-off of 90%, specificity was 85% by sDNA. Detection rate of CRC in the test set was 78%, 64% for adenomas > 1 cm, and 48% for adenomas ≥ 1 cm. Adenoma detection rates were 54% for adenomas > 1 cm, 63% for adenomas ≥ 1 cm, 77% for those > 2 cm, 86% when > 3 cm, and 92% for those > 4 cm. The detection rate for high-grade dysplasia was 69% and 76% for comparably sized adenomas with low-grade dysplasia. The degree of dysplasia did not influence detection rate after being adjusted for size. Similar to adenoma size, the authors found significantly increased CRC detection rates with increased size (P = 0.008). However, when the neoplasm was plotted against quantitative sDNA test scores, there was no significant difference between CRC and adenomas. This study was the first to show that sDNA detection rates increase with the size of adenoma, a finding that is an important factor in screening, as CRC tend to grow in size [13].

**Table 3:** sDNA Sensitivity in the Detection of CRC and Adenomas Examination of sDNA sensitivity in the detection of CRC and adenomas among the training and test sets in a study performed by Aliquist, et al. of 678 patients that had CRC or at least one colorectal adenoma > 1 cm. The training set had a modeled specificity of 90%. The test set had a modeled specificity of 90%; however, it was observed at 85%. sDNA: stool DNA testing; CRC: colorectal cancer

	sDNA Sensitivity	
	Training set	Test set
Colorectal cancer	89%	78%
Adenoma > 1 cm	62%	64%
Adenoma ≥ 1 cm	56%	48%

A 2014 cross-sectional study was performed by Imperiale, et al. which compared 9,989 average risk individuals between the ages of 50 - 84 from June 2011 to November 2012 (Table 4) [12]. All individuals were asymptomatic without a family history of CRC. These individuals also had no personal history of CRC, IBD, or digestive cancers. Patients were not blinded in the study because all patients underwent the same treatment. Patients submitted a stool sample for FIT and sDNA testing, then underwent colonoscopy. Ninety laboratories were used for stool analysis and laboratory personnel were blinded. Patients who dropped out of the study were accounted for, but not evaluated. Patients who could not be evaluated either withdrew consent, did not undergo colonoscopy, or did not submit a stool sample. The study determined that FIT detected 48 of 65 colon cancers, giving it a sensitivity of 73.8% and specificity of 96%. sDNA testing detected 60 of 65 colon cancers and had a sensitivity of 92.3% and specificity of 90% sDNA testing detected 321 of 757 (42.4%) advanced precancerous lesions, whereas FIT detected 180 of 757 (23.8%) of advanced precancerous lesions. Colonoscopy was the reference standard in this survey against which both FIT and sDNA testing were compared. This study determined that 154, 166, and 208 individuals would have to be screened by colonoscopy, sDNA, and FIT, respectively to detect one colorectal cancer of any stage. 166, 178, and 227 individuals would have to be screened by colonoscopy, sDNA, and FIT, respectively, to detect one colorectal cancer of Stage I to III. Lastly, 13, 31, and 55 individuals would have to be screened by colonoscopy, sDNA, and FIT, respectively, to detect one advanced precancerous lesion [12].

**Table 4:** sDNA and FIT Sensitivity in the Detection of CRC and Advanced Precancerous Lesions Comparison of FIT and sDNA testing in the detection of CRC and advanced pre-cancerous lesions in a 2014 study performed by Imperiale, et al., which compared 9,989 average risk individuals between the ages of 50- 84. sDNA: stool DNA testing; FIT: fecal immunochemical test; CRC: colorectal cancer

	Testing Method: Detection and Sensitivity	
	FIT	sDNA
Colorectal cancer (n = 65)	48 (73.8)	60 (92.3)
Advanced precancerous lesions (n = 757)	29 (3.8)	170 (22.5)

A study in 2014 by Heigh, et al. compared sDNA testing against FIT testing in the detection of SSPs > 1 cm (Table 5) [6]. The study utilized a single stool sample of 456 asymptomatic individuals prior to colonoscopy for analysis. Of the 456 individuals, 29 individuals were noted to have SSPs > 1 cm, as well as 232 patients who had no neoplastic findings and were included as controls. Results of the study showed that methylated bone morphogenetic protein 3 (mBMP3) had a significantly higher sensitivity detection rate of SSPs as compared to FIT-50 (50 ng hemoglobin/mL) 66% as compared to 10%, respectively. Specificities were matched at 91%, p = 0.0003. Furthermore, results of the study showed that mBMP3 had a significantly higher sensitivity detection rate of SSPs as compared to FIT-100 (100 ng hemoglobin/mL) 63% as compared to 0%, respectively. Specificities were matched at 95%, p < 0.001. The study found that for the detection of SSPs > 1 cm, the other DNA sequences in the sDNA testing, including NDRG4, mutant KRAS, and β-actin, did not provide any increase in sensitivity as compared to FIT. Finally, this study did not recommend using FIT in the detection of SSPs > 1 cm as FIT-50 and FIT-100 were not found to have high sensitivities during this study [6].

**Table 5:** sDNA, FIT-50, FIT-100 Sensitivities in the Detection of Sessile Serrated Polyps Greater Than 1 cm Comparison of sensitivities of the FIT-100, FIT-50, and sDNA tests in the detection of sessile serrated polyps > 1 cm. This study performed in 2014 by Heigh, et al. [6] obtained single stool samples from 456 asymptomatic individuals prior to colonoscopy for examination. sDNA: stool DNA testing; FIT: fecal immunochemical test

	Testing Method Sensitivity^a^	
	FIT-100	sDNA
Sessile serrated polyps > 1 cm	0%	63%
	Testing method sensitivity^b^	
	FIT-50	sDNA
Sessile serrated polyps > 1 cm	10%	66%
^a^Specificity cutoff was set to 95%. ^b^Specificity cutoff was set to 91%		

In summary, multiple studies showed high sensitivities of sDNA in detecting CRC, at 92.3% and 89%, respectively, when compared with colonoscopy. When comparing sDNA against FIT alone, FIT was consistently less sensitive than sDNA in CRC detection. gFOBT was also found to be less sensitive than sDNA, detecting 14% of CRC, as compared to sDNA, which detected 40% of CRC in the 2004 Imperiale, et al.* *study [4]. Additionally, the Heigh, et al. [6] study found that sDNA testing had greater sensitivity (66% vs 10%) in detecting sessile serrated polyps (SSPs) > 1 cm vs the FIT-50 tests. A significant difference was found when comparing sDNA test to FIT-10. sDNA testing detected 63% SSPs, whereas FIT-100 testing did not detect any SSPs. Overall, sDNA had higher sensitivity than FIT-50, FIT-100, and gFOBT in the detection of adenomas and SSPs. 

## Conclusions

This review discussed multiple methods of colorectal cancer screening. Colonoscopy has been the gold standard of CRC screening for many years, but many patients decline colonoscopy screening due to its invasive nature. To ensure that some form of CRC screening is available in populations denying colonoscopy screening, an alternative screening method with similar sensitivity needs to be utilized. sDNA testing has been shown to have higher sensitivities when compared to the other non-invasive studies available (FIT and gFOBT) in the detection of both cancers and pre-cancerous lesions. However, sDNA is not as sensitive as a colonoscopy for CRC screening. sDNA testing is a useful modality in average-risk adults aged 50 to 75 who prefer non-invasive screening. The United States struggles annually to meet CRC screening goals largely due to the invasiveness of current CRC screening. In patients who prefer a non-invasive screening method, sDNA testing can be used at home as an effective screening tool. The widespread implementation of sDNA could result in higher compliance among US adults for CRC screening. sDNA should also reduce healthcare costs through its ability to identify precancerous and cancerous lesions in patients who otherwise would have refused screening and likely would have presented at an advanced stage.

CRC screening is recommended for all adults between the ages of 50 and 75. Colonoscopy is still the preferred method for CRC screening due to its high sensitivity in CRC detection, the ability for direct visualization, and the ability to physically remove discovered polyps. In patients who refuse colonoscopy or cannot tolerate colonoscopy, sDNA testing should be recommended as a strong alternative. However, given that sDNA is only a screening tool, a positive sDNA test should be followed by a diagnostic colonoscopy. In comparison to colonoscopy, sDNA is less sensitive but has a higher rate of CRC detection when compared to the other non-invasive methods of CRC screening, including FIT and gFOBT. Due to these findings, more physicians should recommend sDNA testing over other non-invasive screening methods in average-risk patients who are between the ages of 50 and 75.

## References

[1] SEER SEER (2016). SEER Stat Fact Sheets: Colon and Rectum Cancer. http://seer.cancer.gov/statfacts/html/colorect.html.

[2] Siegel RL, Miller KD, Jemal A (2016). Cancer statistics, 2016. CA Cancer J Clin.

[3] US Preventive Services Task Force, Bibbins-Domingo K, Grossman DC, Curry SJ, Davidson KW, Epling JW Jr, García FA, Gillman MW, Harper DM, Kemper AR, Krist AH, Kurth AE, Landefeld CS, Mangione CM, Owens DK, Phillips WR, Phipps MG, Pignone MP, Siu AL (2016). Screening for colorectal cancer: US Preventive Services Task Force recommendation statement. JAMA.

[4] Imperiale TF, Ransohoff DF, Itzkowitz SH, Turnbull BA, Ross ME; Colorectal Cancer Study Group (2004). Fecal DNA versus fecal occult blood for colorectal-cancer screening in an average-risk population. N Engl J Med.

[5] Dhaliwal A, Vlachostergios PJ, Oikonomou KG, Moshenyat Y (2015). Fecal DNA testing for colorectal cancer screening: Molecular targets and perspectives. World J Gastrointest Oncol.

[6] Heigh RI, Yab TC, Taylor WR, Hussain FT, Smyrk TC, Mahoney DW, Domanico MJ, Berger BM, Lidgard GP, Ahlquist DA (2014). Detection of colorectal serrated polyps by stool DNA testing: comparison with fecal immunochemical testing for occult blood (FIT). PLoS One.

[7] Heitman SJ, Ronksley PE, Hilsden RJ, Manns BJ, Rostom A, Hemmelgarn BR (2009). Prevalence of adenomas and colorectal cancer in average risk individuals: a systematic review and meta-analysis. Clin Gastroenterol Hepatol.

[8] Jass JR (2001). Serrated route to colorectal cancer: back street or super highway?. J Pathol.

[9] Rex DK, Ahnen DJ, Baron JA, Batts KP, Burke CA, Burt RW, Goldblum JR, Guillem JG, Kahi CJ, Kalady MF, O'Brien MJ, Odze RD, Ogino S, Parry S, Snover DC, Torlakovic EE, Wise PE, Young J, Church J (2012). Serrated lesions of the colorectum: review and recommendations from an expert panel. Am J Gastroenterol.

[10] Young GP, Symonds EL, Allison JE, Cole SR, Fraser CG, Halloran SP, Kuipers EJ, Seaman HE (2015). Advances in fecal occult blood tests: the FIT revolution. Dig Dis Sci.

[11] Pickhardt PJ (2016). Emerging stool-based and blood-based non-invasive DNA tests for colorectal cancer screening: the importance of cancer prevention in addition to cancer detection. Abdom Radiol (NY).

[12] Imperiale TF, Ransohoff DF, Itzkowitz SH, Levin TR, Lavin P, Lidgard GP, Ahlquist DA, Berger BM (2014). Multitarget stool DNA testing for colorectal-cancer screening. N Engl J Med.

[13] Ahlquist DA, Zou H, Domanico M, Mahoney DW, Yab TC, Taylor WR, Butz ML, Thibodeau SN, Rabeneck L, Paszat LF, Kinzler KW, Vogelstein B, Bjerregaard NC, Laurberg S, Sørensen HT, Berger BM, Lidgard GP (2012). Next-generation stool DNA test accurately detects colorectal cancer and large adenomas. Gastroenterology.

